# Internet use, physical activity, and cognitive function in Chinese older adults: a cross-lagged panel analysis

**DOI:** 10.3389/fnagi.2025.1579874

**Published:** 2025-05-08

**Authors:** Jinfu Wang, Na Zhang, Chengxin Huang, Qinmei Wu, Jin Tong

**Affiliations:** ^1^School of Physical Education, South China University of Technology, Guangzhou, China; ^2^School of Nursing, Health Science Center, Xi’an Jiaotong University, Xi’an, China; ^3^School of Physical Education, Shandong University, Jinan, China; ^4^Honghui Hospital, Xi’an Jiaotong University, Xi’an, China

**Keywords:** bidirectional relationship, physical activity, cognitive function, internet use, mediation model

## Abstract

**Background:**

With the arrival of an aging society, cognitive health in older adults has become a global focal point. Cross-sectional studies have shown that internet use and physical activity may significantly affect cognitive function in older adults, but their longitudinal relationships and underlying mechanisms have not been fully explored.

**Objective:**

This study aims to explore the relationship between internet use, physical activity, and cognitive function, and examine the mediating role of physical activity.

**Methods:**

This study uses two rounds of longitudinal data from the China Health and Retirement Longitudinal Study (2018 and 2020), including a total of 2,383 individuals aged 60 and above. Cross-lagged regression analysis is used to test the bidirectional relationship between internet use and cognitive function, while a semi-longitudinal mediation model is used to examine the mediating role of physical activity.

**Results:**

The results indicate that there is a bidirectional relationship between internet use, physical activity levels, and cognitive function. Higher levels of internet use are associated with better cognitive function, and physical activity levels mediate the longitudinal relationship between internet use and cognitive function in older adults.

**Conclusion:**

This study reveals the complex relationship between cognitive function, internet use, and physical activity in older adults, and provides new perspectives for interventions aimed at improving cognitive health in older adults. Future research should further explore the dynamic changes between these variables to develop more effective intervention strategies and improve cognitive health and well-being in older adults.

## 1 Introduction

The rapid acceleration of China’s aging society has led to cognitive decline becoming a critical issue in public health. Research indicates that older adults are at a heightened risk of cognitive decline, which not only exacerbates the prevalence of chronic conditions such as cardiovascular diseases and hypertension but also accelerates the further deterioration of cognitive function (Fang and Zhang, 2024; Gorelick et al., 2011; Fratiglioni et al., 2004). Moreover, cognitive impairment is strongly linked to a heightened risk of mortality (Chen et al., 2024), increased social isolation (Pinquart and Sörensen, 2003), and a greater economic burden on older adults (Hurd et al., 2013). Consequently, safeguarding cognitive health among older adults is essential for mitigating the challenges posed by the aging population.

In the context of the rapid advancement of digital and smart technologies, the internet has emerged as a vital platform for older adults to access information and engage in social interactions, sparking significant attention in the public health domain due to its potential effects on cognitive function. Research suggests a complex, bidirectional relationship between internet use and cognitive function among older adults. The ecological model highlights the challenges that technological advancements present to older adults with limited cognitive resources (Lawton, 1989), underscoring the pivotal role of cognitive function in technology use, particularly for those with diminished cognitive capacities who encounter greater difficulties navigating the internet (Slegers et al., 2009). As individuals age, they naturally experience declines in cognitive functions such as processing speed, attention regulation, and working memory, which may serve as barriers to effective internet use (Hawthorn, 2000; Charness and Boot, 2009). Specifically, older adults with lower levels of fluid and crystallized intelligence are less likely to engage with the internet (Ownby et al., 2008). However, declines in cognitive function do not uniformly affect older adults’ ability to use the internet. Indeed, specific cognitive functions, including memory, domain knowledge, spatial abilities, and psychomotor speed, are strongly correlated with older adults’ success in performing internet-related tasks (Ownby et al., 2008). Strengths in these cognitive functions are significantly associated with higher internet proficiency (Umemuro, 2004), indicating that older adults, even with limited cognitive resources, can effectively use the internet by leveraging their strengths in these cognitive domains.

Existing literature reveals considerable variations in the effects of internet use on cognitive function among older adults. Several studies suggest that internet use is positively associated with enhanced cognitive function in older adults, with more frequent usage being correlated with improved cognitive performance. For example, research by Ordonez et al. (2011) demonstrates that elderly internet users exhibit marked improvements in cognitive function, particularly in language and memory domains. Chan et al. (2016) further found that the use of tablets enhances elderly individuals’ episodic memory and processing speed. A study by Klusmann et al. (2010) offered a 6-month internet training program, which led to significant improvements in both immediate and delayed recall among elderly internet users. However, contrasting studies report that a 12-month internet training program did not yield cognitive benefits for elderly participants (Slegers et al., 2008). Similarly, research involving elderly individuals with mild cognitive impairment (Djabelkhir et al., 2017) or cognitively healthy individuals residing in communities has also reported negligible results (Slegers et al., 2009). Firth et al.’s (2024) research further underscores the dual nature of information technology, highlighting that while prolonged internet use may contribute to cognitive decline, enhancing internet skills can yield significant cognitive benefits and improve overall cognitive function among the elderly. The discrepancies observed in the aforementioned studies suggest the reciprocal association and the underlying processes between internet use and cognitive function in older adults require more in-depth exploration.

In the process of investigating the connection between internet use and cognitive function among older adults, it is of vital importance to reveal the underlying processes. In contemporary Chinese society, with the increase in public health awareness and the widespread promotion of national fitness programs, the interaction between internet use, physical activity, and cognitive function has garnered significant attention. Several studies suggest that physical activity may act as a mediator in the relationship between internet use and cognitive function. A recent review by Firth et al. (2024) posits that excessive internet use, leading to sedentary behavior, may replace time spent on physical activity, thereby serving as a potential pathway for internet use to negatively impact cognitive function. Conversely, Kearns’ study demonstrates that internet use can promote engagement in physical activity through various channels, which, in turn, can positively influence cognitive function (Kearns and Whitley, 2019; James et al., 2023). Although the association between physical activity and cognitive function in older adults is well-established, whether internet use has a positive or negative impact on physical activity remains inconclusive in current studies and necessitate further investigation.

### 1.1 The present study

Overall, the association between internet use, physical activity, and cognitive function is multifaceted, characterized by complexity and variability. In light of the discrepancies in existing research findings, several key issues require further exploration: First, the inconsistencies across studies regarding the impact of internet use on cognitive function in older adults underscore the necessity for a more precise understanding of the relationship between internet use and cognitive function in this population. Second, while a reciprocal relationship between internet use and cognitive function may exist, most studies utilizing cross-sectional designs hinder our ability to establish the bidirectional links between these variables in the context of older adults. Third, although research has explored the potential link between internet use, physical activity, and cognitive function in older adults, the role of physical activity as a mediator in this relationship still requires further empirical verification. To address these gaps, the current study utilizes a 2-year cross-lagged analysis model to overcome the limitations of existing research, probing into the complex dynamic interplay among internet use, physical activity, and cognitive function in older adults. This approach aims to provide robust scientific evidence for developing targeted interventions to promote cognitive health and enhance the quality of life for older adults. Based on a comprehensive review of the above literature, the present study proposes the following hypotheses: (1) internet use has a positive impact on cognitive function in older adults, with this effect exhibiting dynamic changes over time; (2) physical activity mediates the relationship between internet use and cognitive function, meaning that internet use indirectly enhances cognitive function by promoting physical activity; (3) there is a bidirectional relationship between internet use and cognitive function, meaning that improvements in cognitive function may also increase the frequency of internet use.

## 2 Materials and methods

### 2.1 Data source

The data for this study are derived from the China Health and Retirement Longitudinal Study (CHARLS), a comprehensive project spearheaded by Peking University that aims to collect detailed microdata on Chinese adults aged 45 and older (Zhao et al., 2014). These data encompass a broad range of variables, including personal information, health status, physical measurements, family structure, economic support, utilization of medical services, insurance status, employment and retirement conditions, economic indicators (such as income, consumption, and personal assets), and community characteristics, thus offering valuable resources for interdisciplinary research on aging. A comprehensive introduction and research design of the CHARLS project can be accessed through the project’s official website at https://charls.pku.edu.cn/.

In this study, we utilized the two most recent waves of CHARLS data, collected in 2018 (T1) and 2020 (T2), with the 2018 survey serving as the baseline (T1). To ensure the accuracy and reliability of our analysis, we conducted rigorous screening of the sample, excluding participants who were lost to follow-up in subsequent surveys (*n* = 8,347), those under the age of 60 (*n* = 2,422), and those with missing or abnormal data for key variables. Specifically, we excluded participants with missing cognitive function information (*n* = 1,577), missing physical activity information (*n* = 290), and missing covariate information, including gender, age, education level, marital status, smoking, alcohol consumption, rural, residence, income, chronic diseases, and depressive symptoms (*n* = 7,450). Ultimately, 2,383 eligible older adults were included in the analysis (Supplementary Figure S1).

### 2.2 Ethical considerations

Prior to the initiation of the CHARLS study, professionally trained investigators provided each participant with a comprehensive explanation of the study procedures, ensuring that all participants signed informed consent forms. All information collected during the survey was strictly confidential, with participant data rigorously safeguarded in accordance with data security and privacy regulations. Furthermore, the Institutional Review Board (IRB) of Peking University conducted a comprehensive ethical review of all phases of the CHARLS study and granted approval (approval number: IRB 00001052-11015).

### 2.3 Measurements

#### 2.3.1 Cognitive function

In the CHARLS database, cognitive function is assessed across two dimensions: episodic memory and cognitive integrity. The episodic memory section comprises immediate recall and delayed recall, in which participants are required to recall 10 words immediately after hearing them and then attempt to recall them again after a delay of 4–10 min. The episodic memory score is calculated as the sum of immediate recall and delayed recall, with the average of the two tests determining the final score, out of a maximum of 10 points. The cognitive integrity section encompasses time orientation, calculation ability, and drawing ability. Time orientation requires participants to accurately identify the current year, month, day, season, and week, with 1 point awarded for each correct answer. The calculation test asks participants to repeatedly subtract 7 from 100 for five iterations, awarding 1 point for each correct answer. The drawing test requires participants to replicate an overlapping five-pointed star pattern, awarding 1 point for successful reproduction (Ding and He, 2021). The cumulative score across all items reflects the participant’s overall cognitive status, with a total score ranging from 0 to 21 points, where higher scores indicate better cognitive function. This cognitive function assessment tool has demonstrated strong reliability and validity in previous studies (Chai et al., 2024).

#### 2.3.2 Physical activity

This study utilizes the short version of the International Physical Activity Questionnaire (IPAQ) from CHARLS to assess the physical activity levels of older adults. The questionnaire consists of seven questions and classifies activity levels into low intensity (e.g., walking), moderate intensity (e.g., brisk walking), and high intensity (e.g., running), based on reported activity time and weekly frequency. Since the time for physical activity in the CHARLS questionnaire is provided as a range, without specific indications, this study refers to previous research (Li et al., 2020) and converts activity time into specific durations in minutes. Activities ranging from 10 min to less than 0.5 h are assigned 30 min; activities ranging from 0.5 h to less than 2 h are assigned 60 min; activities ranging from 2 h to less than 4 h are assigned 180 min; and activities lasting 4 h or more are assigned 240 min. Metabolic equivalent (MET) is employed as the unit to measure physical activity, with the formula: MET = MET value × weekly frequency (days/week) × daily time (minutes/day). The MET values for different intensity activities are as follows: low intensity = 3.3, moderate intensity = 4.0, and high intensity = 8.0. The MET values of the three intensities are summed, and based on the individual’s physical activity level, participants are classified into three groups: low physical activity group (METs/week < 600 = 1), moderate physical activity group (600 ≤ METs/week ≤ 3,000 = 2), and high physical activity group (METs/week > 3,000 = 3). Physical activity frequency is calculated by summing the number of days for low, moderate, and high intensity activities, with a score range from 0 to 21. The assessment of physical activity and its frequency has shown strong reliability and validity in previous studies (Huang and Lu, 2024).

#### 2.3.3 Internet use

In the CHARLS 2018 and 2020 surveys, the method for measuring internet usage is as follows: First, participants are asked whether they have accessed the internet in the past month to identify internet users. For participants answering “yes,” further questions inquire about the types of devices used for internet access, including desktop computers, laptops, tablets, mobile phones, and other devices. The device diversity score is computed by counting the number of device types reported by each participant. For example, using only a mobile phone assigns 1 point, using both a mobile phone and a computer assigns 2 points, and so forth. The score range is from 0 to 5, with higher scores indicating a greater diversity of devices used for internet access. Since the 2020 data lack detailed information on internet usage frequency, device diversity serves as a proxy indicator for internet usage level. Device diversity reflects both the extent and the habitual nature of internet access and is closely related to an individual’s digital literacy. Mastery of various devices reflects a strong capacity to adapt to different internet environments and access information, which constitutes an important dimension in measuring internet usage level. The method for measuring internet use levels has shown strong reliability and validity in previous studies (Li et al., 2025).

#### 2.3.4 Control variables

In this study, we comprehensively account for potential confounding factors related to elderly internet use, physical activity, and cognitive outcomes. Drawing on existing research, we controlled for the potential impact of sociodemographic characteristics, health behaviors, and health outcomes on elderly internet use, physical activity, and cognitive function (Li et al., 2023; Liu et al., 2024). Sociodemographic variables include age, gender (1 = male, 2 = female), place of residence (1 = urban, 2 = rural), marital status (1 = married, 2 = unmarried), education level (1 = elementary school or below, 2 = middle school, 3 = high school or vocational school, 4 = college or above), household registration (1 = agricultural, 2 = non-agricultural, 3 = unified resident), and personal income (1 = has income, 2 = no income). Health behavior-related variables include smoking status (1 = currently smoking, 2 = previously smoked, 3 = never smoked), drinking status (1 = drinks, 2 = does not drink), and social participation. Health outcome factors include depression levels and number of chronic diseases (1 = no chronic disease, 2 = 1 chronic disease, 3 = at least 2 chronic diseases). To minimize model complexity, all covariates were analyzed using data from 2018.

### 2.4 Statistical analysis

First, we employed means (standard deviations) to describe continuous variables and presented categorical variables using frequencies (percentages) to offer a comprehensive overview of the sample’s general characteristics. Subsequently, Pearson correlation analysis was conducted to evaluate the relationships between key variables. In order to examine the complex interplay among internet use, physical activity, and cognitive function in older adults, we constructed a cross-lagged model, incorporating factors such as age, gender, place of residence, marital status, education level, household registration, individual income, smoking, drinking, social participation, depression levels, and chronic diseases as covariates. In analyzing the potential mediation mechanism, we employed a semi-longitudinal mediation analysis approach. This approach, grounded in the semi-longitudinal mediation design proposed by Cole and Maxwell (2003), enabled us to infer mediating variables and uncover prospective associations between the independent variable (internet use) and the mediator (physical activity), as well as between the mediator and the dependent variable (cognitive function) using only two waves of data. In comparison to traditional cross-sectional data, two-wave longitudinal data established a clear temporal sequence, thereby significantly enhancing the precision of mediation effect testing. During the analysis, we concentrated on the coefficients of two key paths: the regression path coefficient a for-internet use at T1 on physical activity at T2, and the regression path coefficient b for physical activity at T1 on cognitive function at T2. Although Cole and Maxwell suggest that the significance of paths a and b alone is sufficient to indicate a significant mediating effect, to more reliably substantiate the mediation effect, we further examined the significance of the product of the paths, a*b, and employed the Sobel test to validate the significance of the path coefficients and their product, thereby ensuring the robustness of the mediation relationship (Little, 2013).

Data analysis was performed using IBM-SPSS 22.0 software along with its supplementary module AMOS. We employed various fit quality indicators, including the Comparative Fit Index (CFI), Standardized Root Mean Square Residual (SRMR), and Root Mean Square Error of Approximation (RMSEA). A model fit is considered good when the *CFI* value exceeds 0.90 and both *SRMR* and *RMSEA* values are below 0.08 (Hooper et al., 2008; Hu and Bentler, 1999).

To ensure the robustness of the research findings, we conducted two sensitivity analyses. First, we included additional variables beyond the confounders already incorporated into the model to better address potential confounding factors. These variables were selected based on prior studies that linked self-rated health (very good, good, fair, poor, very poor), physical disability, and bodily pain to physical activity or cognitive function (Carr et al., 2025; Oliveira et al., 2025). Second, we used multiple imputation to address missing data. First, we calculated the proportion of missing data for key variables and analyzed their distribution. The results indicated that the proportion of missing data for key variables was below 10% and predominantly associated with certain socioeconomic indicators. To evaluate the randomness of the missing data, we performed Little missing data for key variables was below 10 mediation relationship king, social participation, depression levels, and chron*p* > 0.05), supporting the rationale for applying multiple imputation. Accordingly, we created five complete datasets using multiple imputation and analyzed each dataset to evaluate result consistency. All tests in the statistical analysis were based on two-tailed *p*-values. A *p*-value less than 0.05 indicates statistical significance, meaning the observed associations or effects are not due to random variation but have practical implications.

## 3 Results

### 3.1 Participant characteristics

Table 1 displays the baseline characteristics of eligible participants (*N* = 2,383) in 2018. The mean age of participants was 69.8 years (SD = 6.75), comprising 660 men and 1,723 women. Approximately 51.4% of older adults had no higher than a primary school education. The majority of older adults resided in rural areas (57.6%), were married (67.9%), and had no income (81.4%). Overall, older adults exhibited low smoking and drinking rates. At the baseline assessment, participants reported an average internet usage of 0.14 h, reflecting minimal engagement with internet-based activities among the elderly. The mean activity level was 2.42, the mean activity frequency was 9.39 days, and the mean cognitive function score was 13.4. Table 1 offers further detailed information regarding the baseline characteristics of the elderly.

**TABLE 1:** Baseline characteristics of participants (*N* = 2,383).

Variables	*N* (%)
Age (years), mean (SD)	69.8 (6.75)
**Gender**	
Male	660 (27.7)
Female	1 732 (72.3)
**Residence**	
Urban	1 010 (42.4)
Rural	1 373 (57.6)
**Marital status**	
Married	1 620 (67.9)
Unmarried	761 (32.1)
**Education level**	
Primary school or below	1 225 (51.4)
Junior high school	679 (28.5)
Senior high school or vocational school	440 (18.5)
College or above	39 (1.60)
**Household registration**	
Rural	1 541 (64.7)
Non-agricultural	798 (33.5)
Unified resident household	44 (1.80)
**Income**	
With income	444 (18.6)
Without income	1 939 (81.4)
**Smoking status**	
Current smoker	58 (2.40)
Former smoker	105 (4.40)
Never smoker	2 220 (93.2)
**Alcohol consumption**	
Drinker	597 (25.1)
Non-drinker	1 786 (74.9)
**Number of chronic diseases**	
No chronic diseases	375 (15.7)
One Chronic disease	545 (22.9)
At least two chronic diseases	1 463 (61.4)
Depression level	7.56 (5.92)
Social participation	2.30 (2.74)
T1 internet use, mean (SD)	0.14 (0.34)
T2 internet use, mean (SD)	0.51 (0.57)
T1 physical activity level, mean (SD)	2.42 (0.68)
T2 physical activity level, mean (SD)	2.40 (0.69)
T1 physical activity frequency, mean (SD)	9.39 (4.48)
T2 physical activity frequency, mean (SD)	9.41 (5.10)
T1 cognitive function, mean (SD)	13.4 (2.74)
T2 cognitive function, mean (SD)	13.5 (2.35)

SD, standard deviation; T1, 2018; T2, 2020.

### 3.2 Pearson correlation analysis

Table 2 presents the Pearson correlation coefficients between internet use, physical activity levels, physical activity frequency, and cognitive function at various time points. The results indicate that physical activity levels and frequencies are positively correlated with cognitive function at both T1 and T2. At T1, physical activity level is significantly positively correlated solely with internet use at T1, whereas at T2, both physical activity level and frequency at T1 and T2 exhibit significant positive correlations with internet use. These correlation results lay the groundwork for the construction of the subsequent cross-lagged model.

**TABLE 2:** The relationship between internet use, physical activity, and cognitive function.

Variables	1	2	3	4	5	6	7	8
1. T1 internet use	1							
2. T2 internet use	0.389***	1						
3. T1 physical activity level	0.041*	0.038	1					
4. T2 physical activity level	0.128***	0.124***	0.298***	1				
5. T1 physical activity frequency	0.098***	0.080***	0.709***	0.243***	1			
6. T2 physical activity frequency	0.112***	0.134***	0.235***	0.702***	0.259***	1		
7. T1 cognitive function	0.205***	0.243***	0.084***	0.159*	0.105***	0.139***	1	
8. T2 cognitive function	0.197***	0.270***	0.088***	0.134***	0.086***	0.143***	0.492***	1

*p < 0.05;

***p < 0.001. T1, 2018; T2, 2020.

### 3.3 Bidirectional relationship between internet use, physical activity, and cognitive function

**FIGURE 1:**
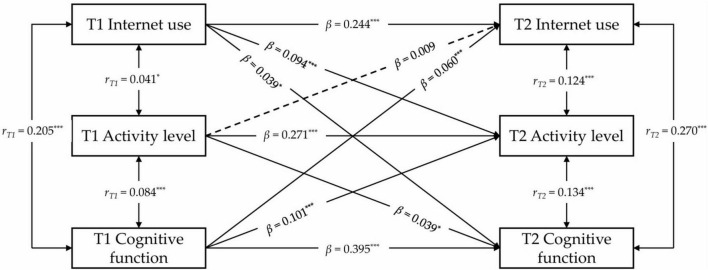
Cross-lagged regression analysis between internet use, activity levels, and cognitive function. *p < 0.05; ***p < 0.001. Where T1 represents the initial measurement time point, and T2 represents the subsequent measurement time point. To simplify presentation, covariates, residuals, residual correlations, and specific observational data are not displayed in this figure. The values marked in this figure represent the standardized regression coefficients for each path. Solid lines indicate statistically significant regression coefficients, while dashed lines indicate non-significant coefficients.

To investigate the associations among internet use, physical activity, and cognitive function, we employed a cross-lagged model while controlling for various covariates, including demographic factors (age, gender, marital status, and education years), self-reported indicators (social status, health, and cognitive ability), as well as chronic conditions, smoking, and alcohol consumption. Initially, the model incorporated internet use, physical activity levels, and cognitive function. The results demonstrated robust model fit (CFI = 0.995, SRMR = 0.007, RMSEA = 0.063) and highlighted significant associations: internet use was positively linked to physical activity levels (*r*_*T*1_ = 0.041, *p* = 0.044; *r*_*T*2_ = 0.124, *p* < 0.001) and cognitive function (*r*_*T*1_ = 0.205, *p* < 0.001; *r_*T*2_* = 0.270, *p* < 0.001) across both time points. Furthermore, physical activity levels were positively associated with cognitive function at both measurement points (*r*_*T*1_ = 0.084, *p* < 0.001; *r*_*T*2_ = 0.134, *p* < 0.001), as illustrated in Figure 1. Regarding directional relationships, internet use at T1 significantly predicted physical activity levels at T2 (β = 0.094, *p* < 0.001), while physical activity levels at T1 did not predict subsequent internet use (β = 0.009, *p* = 0.553). Similarly, higher internet use at T1 was associated with improved cognitive function at T2 (β = 0.039, *p* = 0.036), and greater cognitive function at T1 led to increased internet use at T2 (β = 0.060, *p* < 0.001). Notably, physical activity levels at T1 significantly influenced cognitive function at T2 (β = 0.039, *p* < 0.001), while cognitive function at T1 also served as a strong predictor of physical activity levels at T2 (β = 0.101, *p* < 0.001).

**FIGURE 2:**
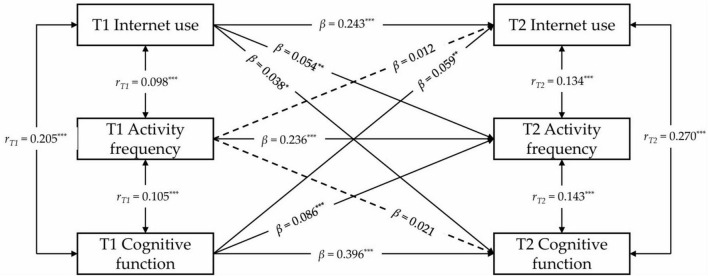
Cross-lagged regression analysis between internet use, activity frequency, and cognitive function. *p < 0.05; **p < 0.01; ***p < 0.001. Where T1 represents the initial measurement time point, and T2 represents the subsequent measurement time point. To simplify presentation, covariates, residuals, residual correlations, and specific observational data are not displayed in this figure. The values marked in this figure represent the standardized regression coefficients for each path. Solid lines indicate statistically significant regression coefficients, while dashed lines indicate non-significant coefficients.

To further examine the interplay between internet use, physical activity frequency, and cognitive function, a cross-lagged model was established, demonstrating satisfactory fit indices (CFI = 0.994, SRMR = 0.008, RMSEA = 0.069). Significant associations were identified between internet use and physical activity frequency at both measurement points (*r*_*T*1_ = 0.098, *p* < 0.001; *r*_*T*2_ = 0.134, *p* < 0.001), as well as between physical activity frequency and cognitive function (*r*_*T*1_ = 0.105, *p* < 0.001; *r*_*T*2_ = 0.143, *p* < 0.001), as illustrated in Figure 2. Analysis revealed that internet use and cognitive function at T1 significantly influenced physical activity frequency at T2 (β = 0.054, *p* = 0.014; β = 0.086, *p* < 0.001). In contrast, physical activity frequency at T1 exhibited no significant predictive effect on either internet use or cognitive function at T2 (β = 0.012, *p* = 0.493; β = 0.021, *p* = 0.238).

### 3.4 Mediating role of physical activity between internet use and cognitive function

**FIGURE 3:**
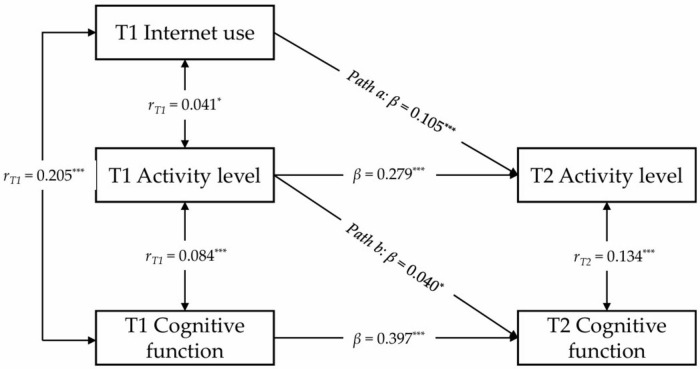
Results of the semi-longitudinal mediation model analysis on the relationship between internet use, activity levels, and cognitive function. *p < 0.05; ***p < 0.001. Where T1 represents the initial measurement time point, and T2 represents the subsequent measurement time point. To simplify presentation, covariates, residuals, residual correlations, and specific observational data are not displayed in this figure. The values marked in this figure represent the standardized regression coefficients for each path. Solid lines indicate statistically significant regression coefficients, while dashed lines indicate non-significant coefficients.

To assess whether physical activity levels and frequency mediate the relationship between internet use and cognitive function in older adults, we developed two semi-longitudinal mediation models. The first model (Figure 3) tested physical activity level as a mediator. The model displayed a strong fit (CFI = 0.993, SRMR = 0.011, RMSEA = 0.072), confirming its suitability for the dataset. Results indicated that both paths a3 and b3 were statistically significant (β = 0.105, *p* < 0.001; β = 0.040, *p* = 0.023), establishing physical activity level as a meaningful mediator in this relationship. To validate these findings, a Sobel test was conducted for path a3b3, yielding a *Z*-value of 2.063 (*p* = 0.019), which further supports the mediating effect of physical activity levels. These results underscore the critical role of physical activity levels in the interplay between internet use and cognitive function over time.

**FIGURE 4:**
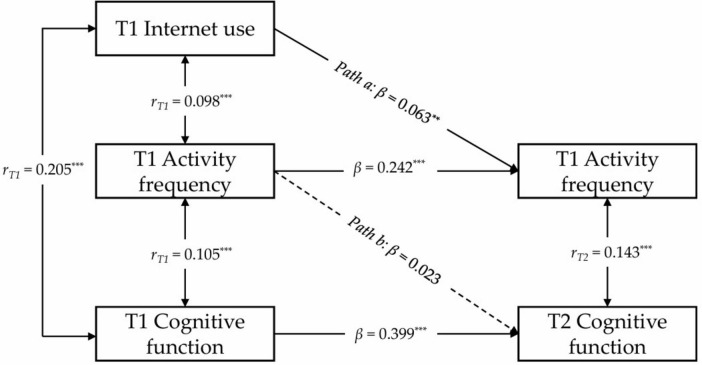
Results of the semi-longitudinal mediation model analysis on the relationship between internet use, activity frequency, and cognitive function. **p < 0.01; ***p < 0.001. where T1 represents the initial measurement time point, and T2 represents the subsequent measurement time point. To simplify presentation, covariates, residuals, residual correlations, and specific observational data are not displayed in this figure. The values marked in this figure represent the standardized regression coefficients for each path. Solid lines indicate statistically significant regression coefficients, while dashed lines indicate non-significant coefficients.

Furthermore, we constructed a semi-longitudinal mediation model (Figure 4) to investigate physical activity frequency as a potential mediator. The model exhibited good fit (CFI = 0.960, SRMR = 0.010, RMSEA = 0.061), suggesting that the model is well-suited to the data. However, path b4 was not statistically significant (β = 0.023, *p* = 0.190), indicating that physical activity frequency at T1 does not significantly predict cognitive function at T2. Consequently, these findings indicate an absence of support for the hypothesis that physical activity frequency acts as a bridge connecting internet use and cognitive function in older adults.

### 3.5 Sensitivity analysis

To ensure the robustness of the research findings, we conducted a sensitivity analysis. Specifically, even when additional variables like self-rated health, physical disability, and bodily pain were included (baseline characteristics of these variables are detailed in Supplementary Table S1), or when missing data were handled using multiple imputation, the main study results remained consistent. This suggests that the associations observed in the initial analysis have strong robustness (Supplementary Figures S2, S3).

## 4 Discussion

As the global elderly population continues to increase, cognitive health has emerged as a critical concern in public health. The rapid advancement of digital technology has not only transformed the lifestyles of the elderly but has also generated considerable interest in the association between internet use and cognitive ability. As a pivotal factor in promoting cognitive health, physical activity has garnered considerable attention from researchers concerning its relationship with internet use and cognitive ability. This study investigates the interactions between internet use, physical activity, and cognitive ability in older adults through a 2-year period of continuous data collection. The results demonstrate that both internet use and physical activity levels in older adults significantly predict cognitive function in a bidirectional manner, with physical activity levels serving as a partial mediator in the relationship between internet use and cognitive function. These findings validate Hypothesis 1 and Hypothesis 3, which proposed that internet use has a positive impact on cognitive function and that there is a bidirectional relationship between internet use and cognitive function.

Consistent with previous studies (Xavier et al., 2014), our research also identified a significant positive correlation between internet use and cognitive function in the older adults. Specifically, increased frequency of internet use is associated with enhanced cognitive function levels among older adults. A comprehensive review of the literature indicates that internet use positively affects health promotion and cognitive function enhancement. Some studies have suggested that regular internet use serves as a protective factor against Alzheimer’s disease among the elderly (Berner et al., 2019). Similarly, frequent internet users among older adults exhibit better cognitive task performance, stronger reasoning abilities, and enhanced memory than their non-user peers (Cho et al., 2023). In general, older adults engage in knowledge acquisition, stimulate creative thinking, and participate in social activities via the internet, which aids in maintaining and enhancing their cognitive function levels. From the perspective of creative thinking, the internet offers a platform for self-expression and artistic creation for older adults, an environment that not only stimulates their brain’s creativity and critical thinking but is also strongly associated with enhanced cognitive function (Firth et al., 2019). Furthermore, the internet provides abundant informational resources and online learning platforms that can stimulate curiosity and foster a desire to learn in older adults, thus promoting the activation and development of cognitive function (Firth et al., 2019). Moreover, cognitive training programs and brain games available online are specifically designed to target different brain regions, such as memory, attention, and processing speed, thereby helping to increase cognitive reserves and mitigate cognitive decline (Anguera et al., 2013). Finally, the internet assists older adults in maintaining connections with the external world. By reading online news and engaging in forum discussions, older adults remain attuned to the surrounding world, preserving cognitive timeliness, which is equally crucial for maintaining cognitive vitality (Xin et al., 2024).

We further investigate the potential mechanisms underlying the impact of internet use on cognitive function in older adults from the perspective of exercise physiology. The results indicate that physical activity levels mediate the relationship between internet use and cognitive function in older adults, consistent with prior research. These findings support Hypothesis 2, which proposed that physical activity mediates the relationship between internet use and cognitive function. The internet, as an information and social platform, lowers the threshold for older adults to engage in physical activity by providing a wealth of fitness information, health guidance, online fitness courses, health apps, and virtual fitness communities, thereby enhancing their health awareness (Quintana et al., 2018; Guo et al., 2022; Zhang et al., 2023). Furthermore, the internet may spark interest in more vigorous physical activities among older adults through personalized exercise prescriptions, fitness challenges, and interactive features within social networks and fitness apps, thereby enhancing enjoyment and motivation for exercise. These factors work synergistically to help older adults increase their physical activity levels, thereby facilitating the achievement of health and fitness goals. Additionally, this study explores the beneficial effects of physical activity on cognitive function. Physiological evidence demonstrates that regular physical exercise can enhance cognitive functions such as memory, attention, and executive function (Cassilhas et al., 2007). Physical exercise exerts a positive influence on cognitive function by enhancing neuroplasticity, stimulating the production of neurotrophic factors, and improving blood circulation (which plays a preventive role against Alzheimer’s disease) (Radak et al., 2010), while also reducing inflammation to protect the brain from inflammation-induced damage (Beavers et al., 2010). According to the cognitive reserve theory, the positive cognitive effects of physical exercise contribute to the enhancement of an individual’s cognitive reserve, thereby helping to prevent cognitive decline and either maintain or improve cognitive function (Erickson et al., 2015; D’Aurizio et al., 2023).

However, when considering physical activity as a mediator, it is crucial to highlight that our research outcomes suggest that the frequency of physical activity in the elderly is not a predictor of their ensuing cognitive function. This may be attributed to the limitations associated with using frequency as a unidimensional indicator. Physical activity frequency focuses exclusively on the count of activities, neglecting other critical factors, such as intensity and duration. Frequency alone may fail to comprehensively capture the multifaceted impact of physical activity on cognitive function. For instance, high-frequency but low-intensity activities may exert limited effects on cognitive function improvement, as cognitive enhancement necessitates a certain threshold of intensity and duration to yield significant outcomes (Zhu et al., 2017). Furthermore, a dose-response relationship exists between physical activity and cognitive function (Loprinzi et al., 2018), suggesting that a minimum threshold of activity is required to yield meaningful effects. Frequency alone may fail to meet this “effective dose,” thereby rendering it ineffective in predicting cognitive function. In contrast, physical activity level, as a more comprehensive indicator, encompasses a range of dimensions, including type, intensity, and duration of activity, thus providing a more holistic perspective on an individual’s activity status and playing a more effective role in predicting cognitive function.

These results elucidate the unidirectional effects on cognitive function in older adults, alongside their underlying mechanisms. Moreover, this study seeks to investigate dynamic and reciprocal relationships among the variables over time. The results confirm the mutual influence between internet use, physical activity, and cognitive function. Specifically, individuals with superior cognitive function are more inclined to extend their online activity time and engage in higher levels of physical activity. This association may reflect their cognitive efficiency in processing online information and managing daily tasks. Regarding exposure to and use of digital technology, older adults with stronger cognitive function demonstrate superior adaptability, which is intricately linked to their cognitive processing speed and efficiency (Berner et al., 2019). As the volume of internet information expands, so too does the cognitive load on older adults. Individuals with superior cognitive function perform more efficiently in filtering and processing online information, whereas those with diminished cognitive function may encounter greater challenges in this process (Torrens-Burton et al., 2017). Furthermore, older adults with superior cognitive function exhibit a reduced perception of information overload, which aids them in maintaining a positive mental state (Zhang and Gao, 2021). They are more likely to adopt a more optimistic attitude toward internet security and possess a heightened sense of trust in the internet, potentially reducing psychological barriers when engaging in online searches (Lu and Wei, 2023). In terms of physical activity, individuals in their advanced years who possess enhanced cognitive capabilities tend to be more committed to a regimen of healthful living., which includes engaging in regular physical activity and sustaining a positive attitude (Cheval et al., 2020). This may be because they are better at planning and executing physical activities and have a deeper understanding of the positive impact of physical activity on health, making them more willing to translate this understanding into action.

### 4.1 Limitations and future directions

A notable strength of this study lies in its utilization of data derived from a nationally representative sample, which furnishes robust data and statistical power, thereby enabling the identification of mediating effects. Furthermore, our research design incorporates a time-series feature, facilitating the exploration of bidirectional relationships between internet usage, physical activity, and cognitive function. Nonetheless, several limitations inherent in this study warrant consideration. Firstly, this study relied on self-reports from participants to assess internet usage, physical activity, and cognitive function, which may have been subject to biases such as social desirability and memory recall, especially concerning physical activity. Previous research has indicated that older adults often overestimate their activity levels when utilizing self-report methods (Meh et al., 2023). Hence, future studies should incorporate more objective measurement tools, such as assessments by healthcare professionals or the use of wearable devices to track activity data, thereby improving both the accuracy and reliability of the data (Wang et al., 2024). Secondly, although we took measures to control for potential confounders, certain unidentified influencing factors may have been inadvertently overlooked. Thirdly, while the cross-lagged model employed in this study elucidates the dynamic relationships between internet usage, physical activity, and cognitive function, it may face limitations in analyzing intra-individual changes and inter-individual differences. Given that the model may not comprehensively account for individual-specific life experiences and environmental factors, it may result in an incomplete explanation of individual differences. Consequently, future research should incorporate more granular individual-level data or employ more advanced statistical methods, such as latent trajectory modeling, to obtain a more nuanced understanding of the complex interactions between these variables. Fourthly, it is important to note that, although our study found an association between internet use and improved overall cognitive function, but this effect may not be consistent across all cognitive domains. Some studies suggest that internet use may impair language fluency (Jin et al., 2009) and short-term memory (Xiong and Yao, 2010), both essential components of cognitive function. This implies that the effects of internet use on cognitive function may be more complex and domain-specific than our findings on overall cognitive function suggest. Future research should investigate the differential effects of internet use on various cognitive domains, such as language ability, to offer a more comprehensive understanding of its impact. This study primarily focuses on the older adult population in China, and sociocultural factors may influence the generalizability of the findings. For example, the sociocultural context in China may shape older adults’ acceptance and usage of the internet, as well as their participation in physical activities. Future research should consider these sociocultural factors to evaluate the applicability of the findings across different cultural contexts. This study used device diversity as a proxy for internet use, which is useful but may not fully capture the frequency or intensity of internet use. Future research should consider additional indicators, such as online time and the frequency of specific online activities, to provide a more comprehensive assessment of internet use among older adults. Additionally, our cross-lagged panel design, while valuable, cannot entirely rule out reverse causation. Specifically, while we observed an association between internet use and improved cognitive function in older adults, we cannot entirely rule out the possibility that older adults with higher cognitive function are more inclined to use the internet, rather than internet use directly enhancing their cognitive function. This potential reverse causation may influence our findings, leading to an overestimation of the benefits of internet use. Future studies could apply advanced statistical techniques, such as instrumental variable regression or Granger causality tests, to further clarify the causal relationship between internet use and cognitive function. Given the significant physiological and psychological impacts of the COVID-19 pandemic on older adults, and considering that our data were primarily collected pre-pandemic, they may not fully reflect post-pandemic changes in cognitive function. In China, after the comprehensive resumption of work following the pandemic, people’s cognitive and emotional patterns have shown marked differences compared to pre-pandemic levels (Duan et al., 2025). These changes may influence the cognitive function of older adults, thereby affecting their relationship with internet use and physical activity. Future studies should explore the long-term effects of the pandemic and the new normal on the cognitive function of older adults, particularly regarding changes in internet use and physical activity. Lastly, cognitive function is a complex phenomenon influenced by various factors across different levels, ranging from physiological foundations at the micro level to socio-cultural influences and environmental conditions at the macro level. Our study primarily focuses on the psychological aspect; future research should strive to incorporate data and methodologies from multiple levels to offer a more comprehensive understanding of cognitive function (Luo et al., 2024). Due to the constraints of the available data, this study incorporates data from only two time points, which limits our capacity to achieve a comprehensive understanding of the dynamic changes between internet usage, physical activity, and cognitive function. Future research could expand the data collection period to more precisely capture the long-term dynamic relationships between internet usage, physical activity, and cognitive function.

## 5 Conclusion and recommendations

In summary, this study, grounded in longitudinal tracking data, investigates the intricate relationships between internet use, physical activity, and cognitive function in older adults, with an emphasis on the mediating role of physical activity. The results indicate that physical activity not only exerts a direct influence on cognitive function in older adults, but also mediates the relationship between internet use and cognitive function. As cognitive status improves, the indirect effect of internet use on cognitive function via physical activity becomes more pronounced. These results underscore the complexity of the interactions among cognitive function, internet use, and physical activity, providing novel insights for interventions designed to enhance cognitive health in older adults. Future research should delve deeper into the dynamic changes in these interactions to develop more effective intervention strategies that promote cognitive health and well-being in older adults.

In light of the positive associations between internet use and physical activity with cognitive health, it is imperative to develop targeted strategies aimed at enhancing internet use and physical activity levels among older adults. With regard to internet use, offering customized online education and social interaction platforms can assist older adults in improving their information processing capabilities and promoting active cognitive function. However, increasing internet use among older adults in China faces several feasibility challenges. First, there is a significant disparity in internet use between urban and rural older adults, with rural seniors having a much lower rate of internet use compared to their urban counterparts (Jing et al., 2025). Second, older adults generally lack digital skills, particularly in device operation, software use, and web navigation (Yoon et al., 2020). Additionally, current internet products are predominantly designed with younger users in mind, often overlooking the specific needs of the middle-aged and elderly populations (Qian et al., 2019). To address these challenges, the government should actively organize offline digital device training courses to disseminate internet knowledge and enhance the digital skills of older adults, thereby narrowing the digital divide between urban and rural areas. Meanwhile, internet companies should accelerate the adaptation of their products for older users by developing interfaces and features that are more user-friendly for this demographic, creating an age-friendly digital environment. Moreover, the government needs to increase investment in internet infrastructure in rural and remote areas, enhancing network coverage to ensure that older adults have equitable access to online resources.

Concerning physical activity, promoting participation in online fitness courses and virtual fitness communities among older adults can reduce barriers to physical activity engagement and foster greater health awareness. However, there are also barriers to physical activity among older adults, such as health conditions, economic constraints, and lack of social support (Harris et al., 2024). Therefore, community and organizational support should provide personalized physical activity plans that consider the health status and interests of older adults to increase their participation. Additionally, it is essential to strengthen the social support system to ensure that older adults have access to necessary resources and support, facilitating the positive psychological and physiological changes resulting from internet use and physical activity, enhancing their role adaptation in daily life, and fostering a robust social support system to address cognitive health challenges.

While our study offers valuable insights into the relationship between internet use, physical activity, and cognitive function in older adults, it is essential to recognize that these variables are part of a complex social structure and may exhibit high-dimensional representational coupling beyond simple linear associations. The complexity of these interactions suggests that future research should consider incorporating representational similarity analysis to examine the cross-temporal or cross-sectional representational similarity of these variables (Luo et al., 2023; Yuan and Luo, 2024; Peng and Luo, 2021), thereby providing deeper insights into the dynamic relationships between internet use, physical activity, and cognitive function in older adults.

## Data Availability

The original contributions presented in the study are included in the article/Supplementary material, further inquiries can be directed to the corresponding author.
