# Supplementary material for: Internet use, physical activity, and cognitive function in Chinese older adults: a cross-lagged panel analysis
Source: Front Aging Neurosci. 2025 May 8;17:1579874. doi: 10.3389/fnagi.2025.1579874 (PMC12095236; doi:10.3389/fnagi.2025.1579874)
Supplement: Supplementary file 1 [file Table_1.doc]

**Multimedia Appendix 1.** Flow of participants into study sample

**
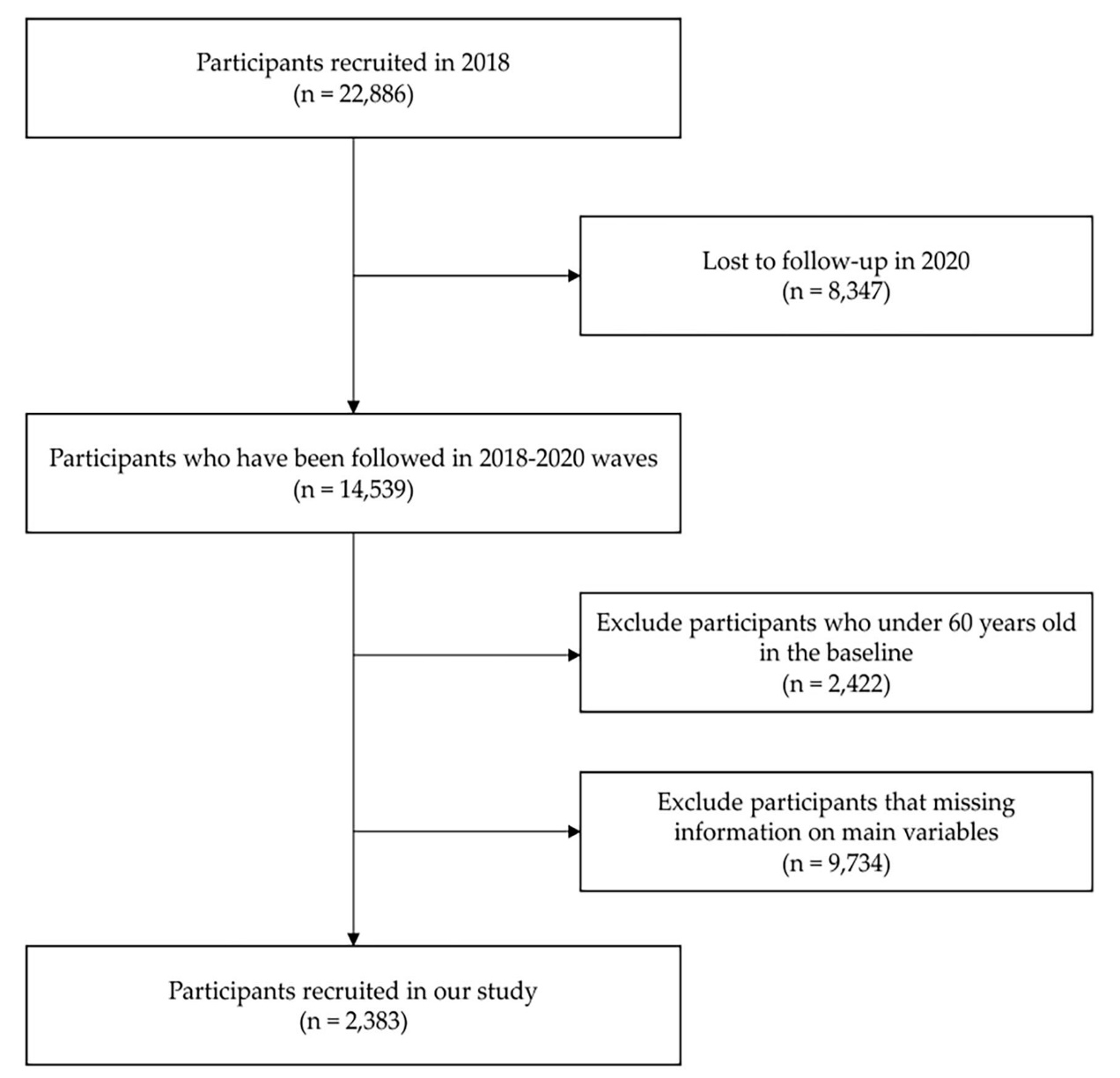
**

**Supplementary Figure S1. Flow of participants into study sample.**

**Multimedia Appendix 2.** Sensitivity analysis with additional control for self-rated health, physical disability, and bodily pain.

**Table S1** Baseline characteristics (self-rated health, physical disability, and bodily pain) of older adults.

| **Variables** | **Mean (SD)/*N* (%)** |
| --- | --- |
| **Self-rated health** | |
| Excellent | 259 (10.9) |
| Good | 321 (13.5) |
| Fair | 1277 (53.6) |
| Poor | 413 (17.3) |
| Very poor | 113 (4.70) |
| **Physical disability** | 18.3 (6.11) |
| **Bodily pain** | 3.05 (3.72) |
| **Household air pollution** | |
| Entirely clean energy use | 100 (2.30) |
| Partially clean energy use | 1912 (44.4) |
| Exclusively solid fuel use | 2292 (53.3) |

**Notes:** SD = standard deviation; ADL = activities of daily living.


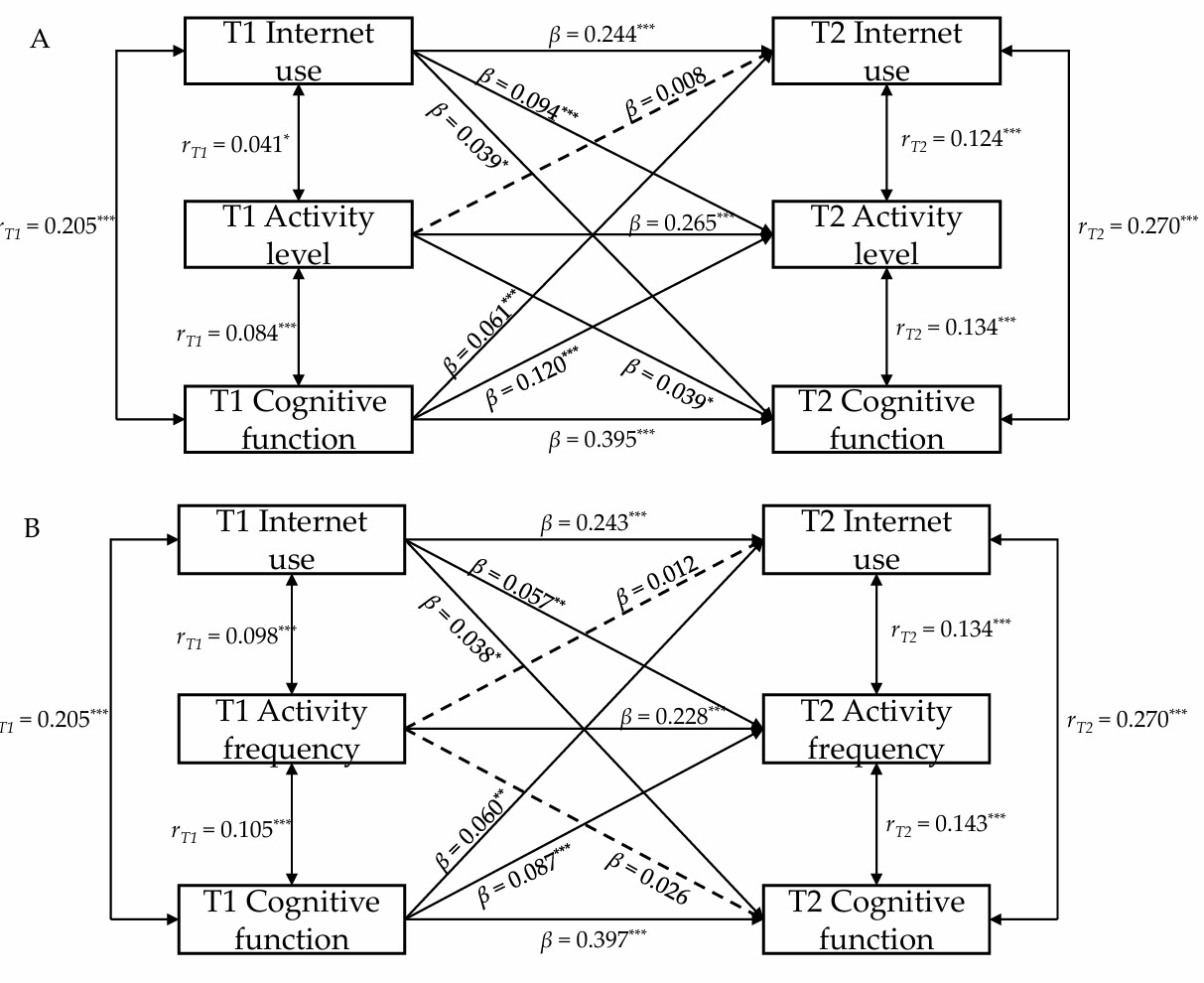

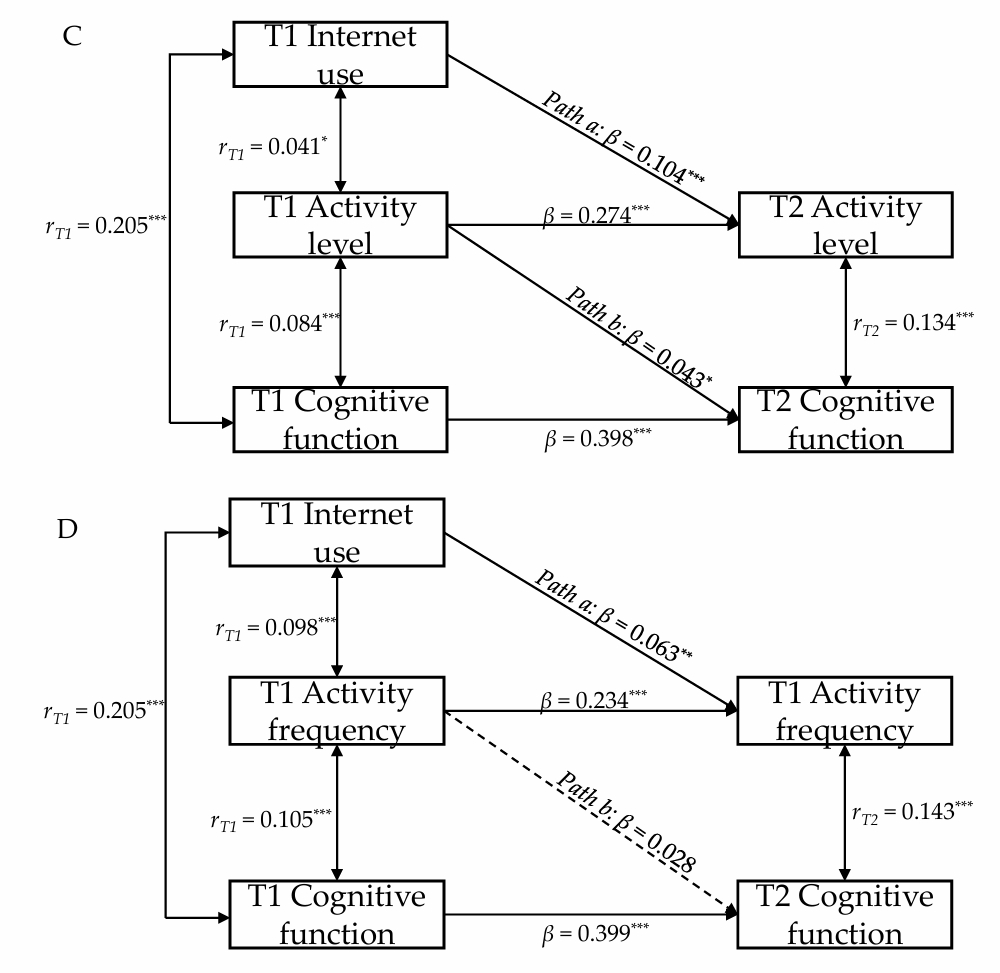


**Figure S2** Cross-lagged regression analysis between internet use, activity, and cognitive function. (A) Cross-lagged regression analysis between internet use, activity levels, and cognitive function. (B) Cross-lagged regression analysis between internet use, activity frequency, and cognitive function. (C) Results of the semi-longitudinal mediation model analysis on the relationship between internet use, activity levels, and cognitive function. (D) Results of the semi-longitudinal mediation model analysis on the relationship between internet use, activity frequency, and cognitive function. To simplify presentation, covariates, residuals, residual correlations, and specific observational data are not displayed in the figure. The values marked in the figure represent the standardized regression coefficients for each path. Solid lines indicate statistically significant regression coefficients, while dashed lines indicate non-significant coefficients. ^**^*p* < 0.01; ^***^*p* < 0.001; β = Standardized coefficient.

**Multimedia Appendix 3.** Sensitivity analysis using multiple imputation for missing data.


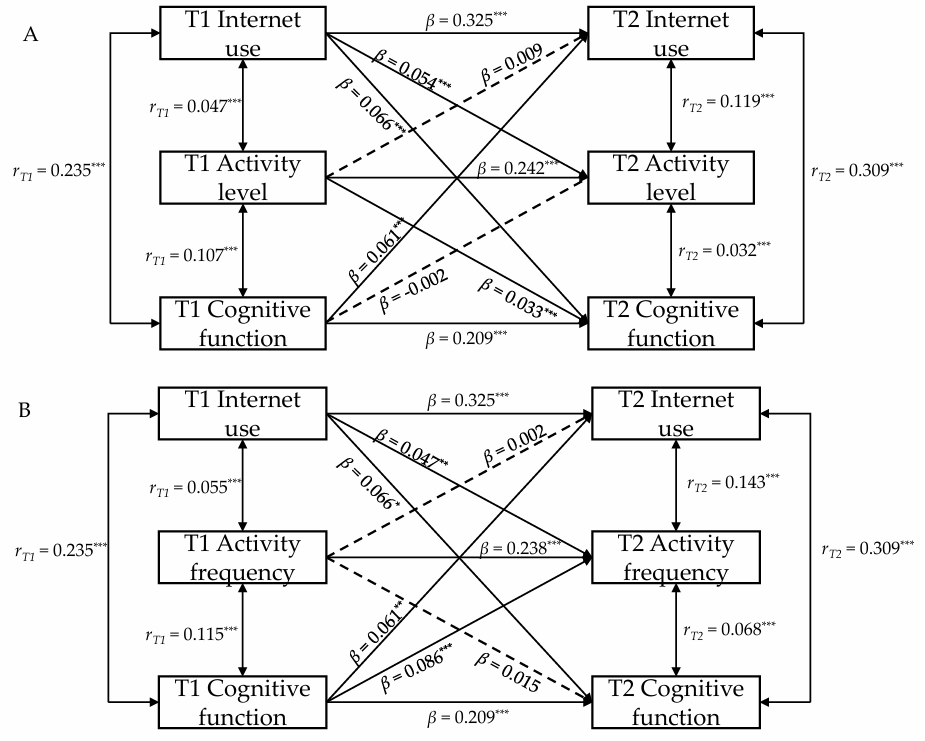

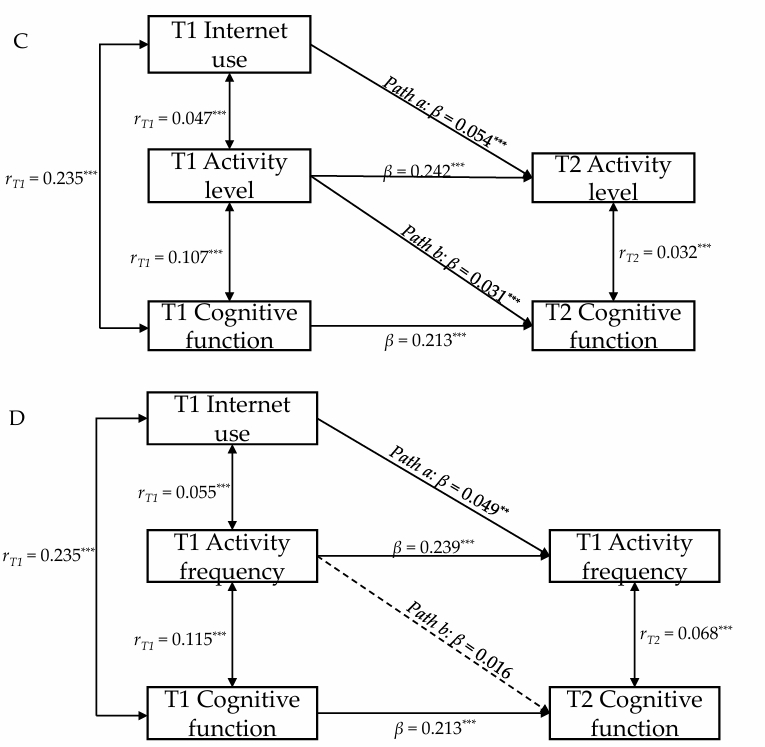


**Figure S3** Cross-lagged regression analysis between internet use, activity, and cognitive function. (A) Cross-lagged regression analysis between internet use, activity levels, and cognitive function. (B) Cross-lagged regression analysis between internet use, activity frequency, and cognitive function. (C) Results of the semi-longitudinal mediation model analysis on the relationship between internet use, activity levels, and cognitive function. (D) Results of the semi-longitudinal mediation model analysis on the relationship between internet use, activity frequency, and cognitive function. To simplify presentation, covariates, residuals, residual correlations, and specific observational data are not displayed in the figure. The values marked in the figure represent the standardized regression coefficients for each path. Solid lines indicate statistically significant regression coefficients, while dashed lines indicate non-significant coefficients. ^**^*p* < 0.01; ^***^*p* < 0.001; β = Standardized coefficient.
